# More than a loading control:Studying the actin cytoskeleton for novel anti-aging mechanisms.

**DOI:** 10.1093/geroni/igaf122.1850

**Published:** 2025-12-31

**Authors:** Maxim Averbukh, Naibedya Dutta, Daniel Hicks, Gilberto Garcia, Ryo Higuchi-Sanabria

**Affiliations:** University of Southern California, Los Angeles, California, United States; University of Southern California, Los Angeles, California, United States; University of Southern California, Los Angeles, California, United States; University of Southern California, Los Angeles, California, United States; University of Southern California, Los Angeles, California, United States

## Abstract

The actin cytoskeleton is a three-dimensional scaffold of proteins that is a regulatory, energy-consuming network with dynamic properties to shape the structure and function of the cell. Proper actin function is required for many cellular pathways, including cell division, autophagy, chaperone function, endocytosis, and exocytosis. Deterioration of these processes manifests during aging and exposure to stress, which is in part due to the breakdown of the actin cytoskeleton. However, the regulatory mechanisms involved in preservation of cytoskeletal form and function are not well understood. Thus, we performed a multi-pronged, cross-organismal screen combining a whole-genome CRISPR-Cas9 screen in human fibroblasts with in vivo C. elegans synthetic lethality screening to identify novel regulators of actin health. We identified the bromodomain protein, BET-1, as a key regulator of actin function and longevity. Overexpression of bet-1 preserves actin function at late age and promotes lifespan and healthspan in C. elegans. These beneficial effects are mediated through actin preservation by the transcriptional regulator function of BET-1. Interestingly, we also find that BRD4 (homolog of bet-1) is required for senescent cell survival. Interestingly, upon neuron-specific overexpression of bet-1, we identified a novel neuron-to-body communication driven by dopamine signaling that extends lifespan by over 70%. This non-autonomous signaling drives peripheral protein homeostasis to drive lifespan in an HSF-1-dependent, but actin-independent mechanism. Together, our discovery assigns a key role for BET-1 in promoting longevity through actin homeostasis when acting autonomously in peripheral tissue, but a unique actin-independent method to promote protein homeostasis when actin non-autonomously through dopaminergic neurons.

